# Arrestins as programmable integrators of GPCR signaling: structural microstates, spatiotemporal logic, and therapeutic control

**DOI:** 10.1038/s41421-026-00909-1

**Published:** 2026-08-25

**Authors:** Qian He, Li-Hua Zhao, H. Eric Xu

**Affiliations:** 1 State Key Laboratory of Drug Research, Shanghai Institute of Materia Medica, Chinese Academy of Sciences, Shanghai, China; 2 University of Chinese Academy of Sciences, Beijing, China; 3 State Key Laboratory of Medical Genomics, Research Center for Medicinal Structural Biology, National Research Center for Translational Medicine at Shanghai, Ruijin Hospital Affiliated to Shanghai Jiao Tong University School of Medicine, Shanghai, China

**Keywords:** Structural biology, Molecular biology

## Abstract

Arrestins were originally defined as terminators of G protein-coupled receptor (GPCR) signaling, yet structural and mechanistic advances now reveal them as programmable, spatiotemporal integrators of cellular signaling. Recent cryo-electron microscopy studies have revealed a diverse spectrum of GPCR–arrestin engagement modes, including core-, tail-, loop-, side-engaged, and membrane-anchored conformations, across GPCR classes and arrestin isoforms. These structures reveal that arrestin recruitment operates as a conditional, allosterically regulated process rather than a binary on–off switch. The selection of the arrestin microstate is governed by layered regulatory inputs, including GPCR kinase-dependent phosphorylation barcodes, membrane and lipid cofactors, and isoform-specific mechanics, which together define the signaling geometry, duration, and subcellular localization. This structural logic provides a mechanistic foundation for biased signaling, noncanonical endosomal signaling, and GPCR-independent arrestin functions. Importantly, emerging therapeutic strategies, including intracellular allosteric modulators and molecular glues, demonstrate that arrestin signaling can be reprogrammed by directly sculpting transducer assemblies rather than ligand efficacy alone. Here, we synthesize recent structural, biochemical, and physiological insights to outline how arrestins decode regulatory inputs into signaling outcomes and how this knowledge enables the development of next-generation, structure-guided GPCR therapeutics.

## Introduction

G protein-coupled receptors (GPCRs) constitute the largest superfamily of cell-surface signaling proteins in mammals, mediating responses to diverse stimuli ranging from photons to peptide hormones and serving as targets for ~36% of approved drugs^1,2^. Traditionally, GPCRs transduce signals via two distinct pathways: G protein activation and arrestin recruitment^3,4^. The arrestin family comprises four isoforms: retina-specific visual arrestins (arrestin-1 and -4) and ubiquitously expressed non-visual arrestins (arrestin-2 and -3, commonly referred to as β-arrestin 1 and 2, or βarr1/2)^5,6^. Once viewed merely as signal terminators, arrestins are now recognized as versatile, spatiotemporal scaffolds that both terminate G protein signaling and initiate distinct pathways (MAPK/ERK, JNK, etc.) and perform noncanonical, GPCR-independent functions^7–14^. This duality underpins the concept of biased (functionally selective) signaling and motivates structure-guided pharmacology.

The landmark crystal structure of the rhodopsin–arrestin-1 complex (PDB: 4ZWJ)^15^ provided the first definitive proof of the core-engaged state, revealing how the finger loop inserts into the receptor’s intracellular bundle to form a high-affinity interface and showing that arrestin engages the membrane directly through its C-edge. Importantly, earlier structural insights into phosphorylation-dependent β-arrestin activation were obtained from the crystal structure of βarr1 bound to a GPCR-derived phosphopeptide, which revealed how clustered phosphates engage basic pockets in the βarr1 N-domain to induce the active conformation^16^. Building on this framework, the concept of the “phosphorylation barcode”^17–20^ — distinct GPCR kinase (GRK)-mediated patterns on receptor C-terminal tails or intracellular loops — was subsequently structurally elucidated in the rhodopsin–arrestin-1 complex (PDB: 5W0P)^21^. This structure revealed the precise coordination of phosphoresidues by the arrestin-1 N-domain, demonstrating how these patterns regulate the binding geometry and activation.

Building on these foundations, the recent “resolution revolution” in cryo-electron microscopy (cryo-EM) has further transformed the field. By utilizing lipid nanodiscs or conformation-selective stabilizers, researchers have moved beyond static snapshots to reveal that arrestin engagement is not a monolithic process but rather a diverse structural continuum dictated by receptor topology and the membrane context^15,21–41^.

This structural “multiverse” spans from the deep core conformation, which sterically blocks G proteins to drive desensitization, to the tail conformation^29,42,43^, which allows simultaneous G protein binding. The specific architecture is tuned by isoform-specific mechanics, such as the distinct C-edge membrane anchoring of βarr1 vs βarr2^44^, and by the receptor class, with class B receptors capable of forming stable “megacomplexes” that drive sustained endosomal signaling^42^. Furthermore, novel “loop-engaged”^33^, “side-engaged”^35^, and membrane-anchored^34^ conformations have been identified in recent structures, indicating that arrestin can decode complex regulatory inputs to adopt highly specialized conformations.

Mechanistically, arrestin recruitment and functional output are encoded by layered regulatory codes: phosphorylation barcodes (e.g., PxPP and PxxPxϕ) together with the receptor’s activation state determine the arrestin binding geometry, conformational activation, and complex stability^16,21,45–47^. Multivalent engagement of receptor-attached phosphates by arrestin phosphate-sensing residues (lysine/arginine) disrupts the interdomain polar core and the three-element autoinhibitory interaction, permitting the transition from the basal to the high-affinity, receptor-bound arrestin conformation^16,48,49^. This information is integrated with a complementary lipid code, in which membrane phosphoinositides such as phosphatidylinositol 4,5-bisphosphate (PIP_2_) and the cofactor inositol hexakisphosphate (IP6) allosterically orient and prime arrestin for receptor engagement and C-edge insertion^50–55^. Together, these regulatory layers define a continuum of arrestin microstates, each with distinct signaling capacities.

These structural and mechanistic insights provide a molecular foundation for biased signaling, in which ligands preferentially stabilize receptor–G protein or receptor–β-arrestin microstates linked to therapeutic signaling while minimizing adverse effects^56–62^. Moreover, the recognition of megacomplex and compartmentalized endosomal signaling has reshaped our understanding of how internalized receptors contribute to physiological processes, from metabolism to neuroplasticity, motivating the development of strategies for generating spatially and temporally targeted therapies^42,63–68^.

Together, these findings establish that GPCR–arrestin coupling spans a continuous structural and functional spectrum shaped by receptor topology, phosphorylation patterns, the lipid environment, and arrestin isoform-specific mechanics. Arrestin recruitment is therefore not a binary on–off switch but a conditional, allosterically regulated process that encodes distinct signaling outputs through discrete conformational microstates. This emerging structural multiverse provides the mechanistic foundation for biased signaling, endosomal signaling, and arrestin-mediated functions. In this review, we synthesize the results from recent high-resolution structural, biochemical, and mechanistic studies to map the diversity of GPCR–arrestin engagement modes, elucidate the molecular logic by which regulatory inputs are decoded, and highlight how these insights enable rational design of biased ligands, intracellular allosteric modulators (IAMs), and arrestin-directed therapeutic strategies.

## The structural landscape of GPCR–arrestin complexes

The transition from X-ray crystallography to cryo-EM has substantially advanced GPCR–arrestin structural biology, enabling the visualization of these dynamic complexes in near-native lipid environments and revealing that their coupling is not a monolithic process but a diverse continuum of engagement modes dictated by the receptor topology and isoform specificity.

### Capture of GPCR–arrestin complexes

GPCR–arrestin complexes were historically more difficult to resolve than GPCR–G protein assemblies because they require receptor phosphorylation and exhibit pronounced structural heterogeneity and interdomain flexibility. Although early crystallographic studies established the foundational core conformation, recent cryo-EM studies have expanded this limited static snapshot into a much broader conformational landscape^15,16,21,22,43,69^. Methodological innovations have driven this progress, significantly expanding the structural repertoire^23–40^ (Table 1). Cryo-EM combined with lipid nanodiscs or lipid layers preserves the membrane contacts essential for arrestin binding^25,44^.

**Table 1:** Structures of the GPCR‒arrestin complex.

Receptor	Arrestin	Technique	Resolution (Å)	PDB	Conformation or other features	Ref.
Rhodopsin	Arr1	XFEL	3.30	4ZWJ	Core	^15^
Rhodopsin	Arr1	XFEL	7.70	5DGY	Core	^22^
Rhodopsin	Arr1	XFEL	3.01	5W0P	Core	^21^
NTSR1	βarr1	Cryo-EM	4.90	6PWC	Core	^23^
NTSR1	βarr1	Cryo-EM	4.20	6UP7	Core	^24^
M2R	βarr1	Cryo-EM	4.00	6U1N	Core	^25^
Turkey β_1_AR	βarr1	Cryo-EM	3.30	6TKO	Core	^26^
V2R	βarr1	Cryo-EM	4.73	7R0C	Core	^27^
5-HT_2B_R	βarr1	Cryo-EM	3.30	7SRS	Core	^28^
GCGR	βarr1	Cryo-EM	3.30 3.50	8JRV 8JRU	Tail Tail and ligand-free	^29^
CB1R	βarr1	Cryo-EM	3.60	8WRZ	Core	^30^
CB1R	βarr1	Cryo-EM	3.20	8WU1	Core	^31^
mGlu3R	βarr1	Cryo-EM	3.70 3.90	9II2 9II3	Core and dimer-2:2 Core and dimer-2:1	^32^
NTSR1	βarr1	Cryo-EM	2.65 3.41	8ZYT 9M0D	Core	^33^
			2.65 2.83	8ZYU 8ZYY	Loop-engaged	
ACKR3	βarr1	Cryo-EM	3.40	9E82	Membrane-anchored	^34^
GPR52	βarr1	Cryo-EM	3.86 3.64	9IJR 9IJS	Side-engaged Side-engaged and ligand-free	^35^
APJR	βarr1	Cryo-EM	3.21 3.49 3.57	9KUV 9KUW 9KUX	Core and monomer Core and dimer-2:2 Core and dimer-2:1	^36^
mGlu8R	βarr1	Cryo-EM	3.14	9MBA	Core and dimer-2:2	^37^
GPR1	βarr1	Cryo-EM	3.30	9UYH	Core conformation 1	^38^
			3.20	9UYI	Core conformation 2	
			3.30	9UYJ	Core conformation 3	
			3.30	9UYL	Core conformation 4	
			2.90	9UYN	Core and ligand-free	
	βarr2		3.50	9UYM	Core conformation 1-like	
MOR	βarr1	Cryo-EM	2.80 2.80	9WSV 9WSX	Core	^39^
PTH1R	βarr1	Cryo-EM	3.07 3.20	9LXR 9LXP	Core	^40^

Core conformations 1–4 denote four distinct core conformations of β-arrestin observed in the GPR1–βarr1 structures.

5-HT_2B_R 5-hydroxytryptamine receptor 2B, CB1R cannabinoid receptor 1, ACKR3 atypical chemokine receptor 3, Arr1 arrestin-1, XFEL X-ray free electron laser.

Pioneering stabilization strategies have been indispensable to overcome the profound inherent flexibility of these assemblies. These approaches include receptor engineering (e.g., appending a hyperphosphorylated surrogate C-tail, such as that of the vasopressin 2 receptor, V2R), direct receptor‒arrestin fusions, the use of pre-activated/truncated arrestins, and the application of conformationally selective binders (e.g., Nb32^70^, scFv30^43^, and Fab7^34^) to trap transient intermediates. While these ingenious tools have profoundly advanced the field, their methodological caveats must be recognized. By design, surrogate tails, arrestin fusions, and rigid chaperones restrict natural interdomain flexibility to funnel the complex toward specific structural outcomes. Moreover, cryo-EM exquisitely captures the most stable, energetically favorable states within a broader dynamic continuum, providing critical high-resolution snapshots of discrete conformational states rather than the complete, transient spectrum of intermediates.

**Fig. 1:**
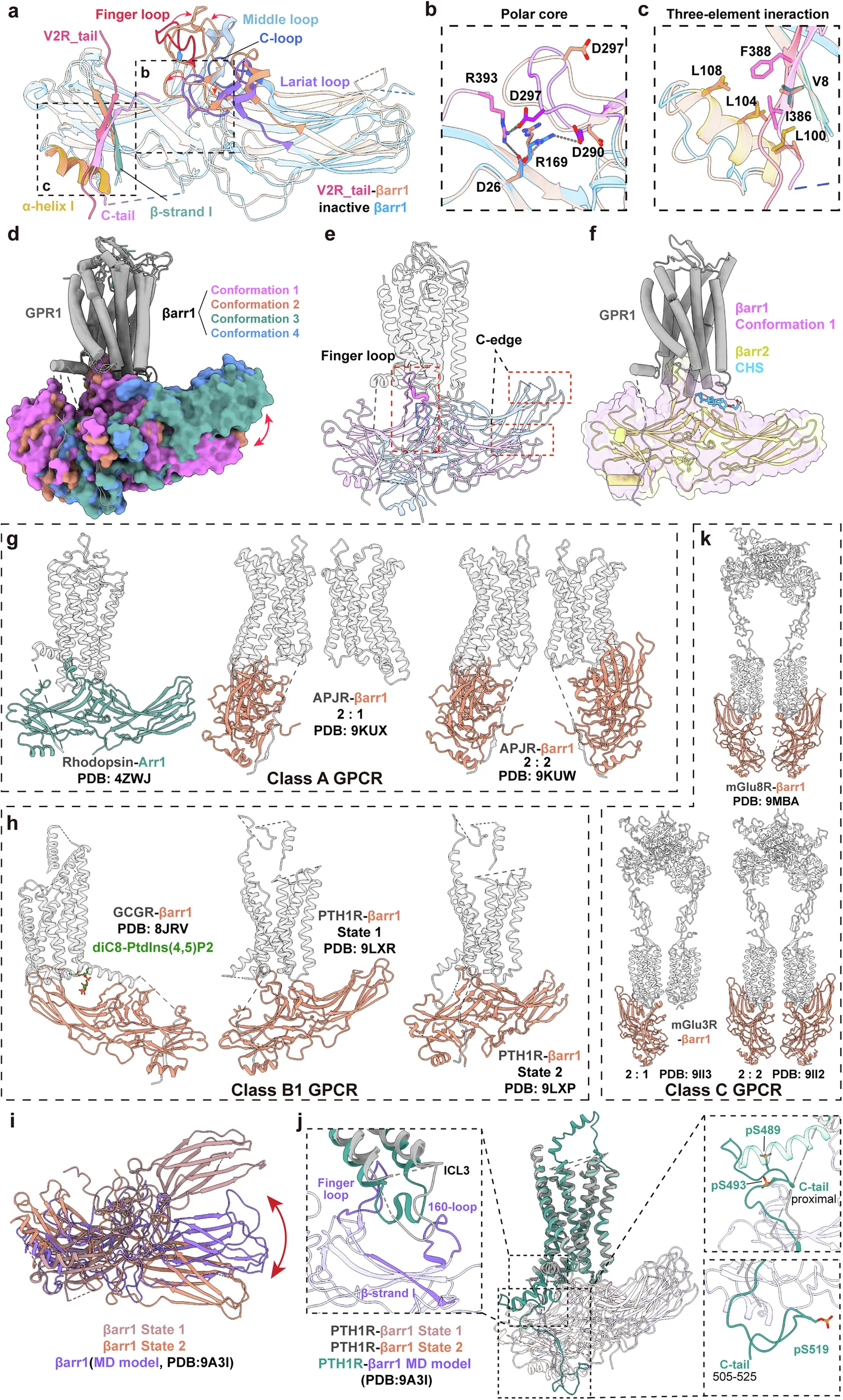
Structural basis of arrestin activation and diversity of GPCR–arrestin interactions. **a** Superposition of the structures of inactive βarr1 (PDB: 1G4M) and active V2R-bound βarr1 (PDB: 7R0C). Hallmark activation features of the coordinated rearrangement of central crest loops are highlighted. **b**, **c** Molecular restraints stabilizing arrestin in the inactive state. Rearrangement of the polar core network upon activation (**b**). Disruption of the three-element interaction (**c**) among the C-terminal tail, β-strand I, and α-helix I after activation. **d** Four distinct arrestin conformations observed in the GPR1–βarr1 complex (PDB: 9UYH, 9UYI, 9UYJ, and 9UYL), illustrating isoform-specific conformational heterogeneity and tilted engagement geometries. **e** Structural comparison of finger loop and C-edge loop configurations across distinct GPR1–βarr1 conformations, highlighting differences in membrane anchoring and engagement depth. **f** Comparison between GPR1–βarr1 (conformation 1) and GPR1–βarr2 (PDB: 9UYM). A cholesteryl hemisuccinate (CHS) molecule that mediates a cholesterol-dependent interaction between GPR1 and βarr2 is shown as sticks. **g** Representative class A GPCR–arrestin assemblies, including rhodopsin–arrestin-1 (left panel; PDB: 4ZWJ) and APJR–βarr1 dimeric complexes exhibiting asymmetric 2:1 (middle panel; PDB: 9KUX) and symmetric 2:2 (right panel; PDB: 9KUW) stoichiometries. **h** Class B1 GPCR–arrestin complexes, including the GCGR–βarr1 complex (left panel; PDB: 8JRV) and two distinct PTH1R–βarr1 conformations (middle and right panels; PDB: 9LXR and 9LXP), illustrating the dynamic equilibrium between tail-engaged and core-engaged architectures. **i**, **j** Comparison of global orientations (**i**) and local interface differences (**j**) between the PTH1R–βarr1 cryo-EM structures and an integrative model derived from live-cell crosslinking (PDB: 9A3I). The crosslinking-guided molecular dynamics model, which was built upon 136 validated intermolecular proximity points, captures highly flexible elements that are invisible in cryo-EM images and reveals a highly heterogeneous conformational ensemble. **k** Class C GPCR–arrestin complexes, including the mGlu8R–βarr1 dimer (top panel; PDB: 9MBA) and two distinct mGlu3R dimeric assemblies (bottom panels; PDB: 9II2 and 9II3), highlighting obligate dimerization, variable stoichiometry, and shallow peripheral arrestin engagement.

Collectively, arrestins share a conserved bilobed architecture composed of N- and C-domains held in the inactive state by two key restraints: a polar core formed by a network of charged residues and a three-element interaction involving the arrestin C-terminal tail, β-strand I, and α-helix I^16,48,49^ (Fig. 1a–c). Engagement with phosphorylated GPCR tails or intracellular loops (especially intracellular loop 3 (ICL3)) disrupts these restraints, releasing the arrestin C-tail and inducing a characteristic interdomain rotation (~18°–25°)^16,45,47,71^. This rearrangement repositions critical functional elements, including the finger, lariat, middle, and C-loops, to enable receptor contact and membrane association^27,43,44,72,73^ (Fig. 1a). Importantly, arrestin activation is best described as conformationally heterogeneous and state-selective rather than strictly on–off.

### Structural features of arrestin isoforms

Comparative structures show that arrestin recruitment is a logic-gated selection process: isoform mechanics, membrane coupling, and the receptor architecture together determine the geometry and function.

Cryo-EM comparisons indicate that isoform selectivity stems from differences in the C-edge length, membrane anchoring, lipid dependence, and conformational flexibility — not a simple core vs tail dichotomy^34,38,44,45,47^. For example, βarr2 displays a significantly larger interdomain rotation (~23°–25°) than βarr1 (~18°–20°)^45,47^. In atypical chemokine receptor 3 (ACKR3), βarr1 forms a tightly membrane-anchored complex with deep finger-loop insertion and robust C-edge penetration, whereas βarr2 contacts the membrane via the finger loop but lacks stable C-edge anchoring because of a shorter loop^34^. Thus, both isoforms interact with the receptor tail and the membrane but differ in membrane coupling efficiency and conformational stabilization, leading to distinct scaffolding outcomes.

In G protein-coupled receptor 1 (GPR1), isoform divergence is reversed in terms of conformational heterogeneity^38^. βarr1 has multiple binding conformations, including tilted, membrane-anchored states supported by its extended C-edge loops^38^ (Fig. 1d, e). In contrast, βarr2 adopts a single, well-defined core-engaged conformation with a non-membrane-anchored C-edge (Fig. 1f). Notably, the GPR1–βarr2 complex is stabilized by a cholesterol-mediated interface bridging ICL2 and βarr2 central loops (Fig. 1f), a feature that is absent in βarr1 complexes and selectively required for βarr2 recruitment and signaling^38^.

### Structural features of arrestin in distinct GPCRs

Beyond isoform effects, GPCR class-specific architectures further dictate arrestin stoichiometry and geometry. Most resolved class A GPCRs typically support the formation of a 1:1 complex with variable degrees of finger-loop penetration^15,21,23–28,30,31,33–36,38,39^ (Fig. 1g). Structures of the apelin receptor (APJR) can assemble asymmetric 2:1 and symmetric 2:2 receptor–βarr1 complexes in which bridging or steric crowding favors tail-dominant or shallow-core engagement^36^ (Fig. 1g).

Class B1 GPCRs exhibit dynamic β-arrestin engagement equilibria. The glucagon receptor (GCGR)‒βarr1 complex adopts a tail-engaged conformation^29^ (Fig. 1h). βarr1 stabilization relies on extensive C-tail phosphorylation and PIP_2_-mediated C-edge insertion, while the transmembrane domain (TMD) core remains accessible for simultaneous G protein binding^29^. This architecture permits megacomplex formation and sustained endosomal signaling. In contrast, parathyroid hormone 1 receptor (PTH1R)–βarr1 structures^40^ (PDB: 9LXR and 9LXP) reveal that class B1 receptors can stabilize both tail-engaged and core-engaged states (Fig. 1h). This structural plasticity enables both sustained endosomal signaling (tail state) and receptor desensitization (core state).

In addition to cryo-EM snapshots, the conformational heterogeneity of the PTH1R–βarr1 interaction has been elucidated through orthogonal live-cell crosslinking^74^. When directly compared to cryo-EM structures, this crosslinking-derived model provides crucial complementary insights. While cryo-EM resolves distinct core-engaged states, the crosslinking data capture a dynamic conformational ensemble^40,74^ (Fig. 1i). Unbiased molecular dynamics simulations, which are guided by these extensive proximity constraints, confirm that this native ensemble dynamically bridges the discrete cryo-EM states through a 13º–25° fluctuation in the βarr1 orientation^74^. Furthermore, this approach revealed flexible elements that are intrinsically invisible in cryo-EM density maps, tracking highly mobile ICL3 and revealing unappreciated interfaces, such as the proximal phosphorylation cluster engaging the arrestin “N-edge” and extended interactions of the distal C-terminal tail with the N-domain^40,74^ (Fig. 1j). Together, integrating cryo-EM with in-cell conformational mapping provides a more complete and mechanistically robust understanding of PTH1R–βarr1 interactions.

Class C GPCRs function as obligate dimers with stoichiometric versatility imposed by the dimer architecture. Unlike class A receptors, β-arrestin-bound metabotropic glutamate receptors (mGluRs) retain transmembrane helix 6 (TM6) in an inward, inactive-like conformation, thereby precluding deep arrestin core insertion^32,37^. As a result, arrestin engages mGluRs through a shallow, peripheral interface rather than through a canonical core pocket (Fig. 1k). The βarr1 finger loop occupies a cavity formed by TM3, ICL1, ICL2, and ICL3, while the primary binding energy is derived from extensive contacts with the phosphorylated C-tail. Metabotropic glutamate receptor subtype 3 (mGlu3R) supports the formation of an asymmetric 2:1 complex, in which a single βarr1 binds one protomer and allosterically stabilizes an asymmetric dimeric unit, whereas both mGlu3R and mGlu8R form symmetric 2:2 assemblies with one βarr1 bound to each protomer^32,37^. In both cases, βarr1 binding favors scaffolding-competent states while excluding the deep-core, desensitization-centric mechanisms characteristic of monomeric class A receptors.

### Engagement conformations: a dynamic continuum

**Fig. 2:**
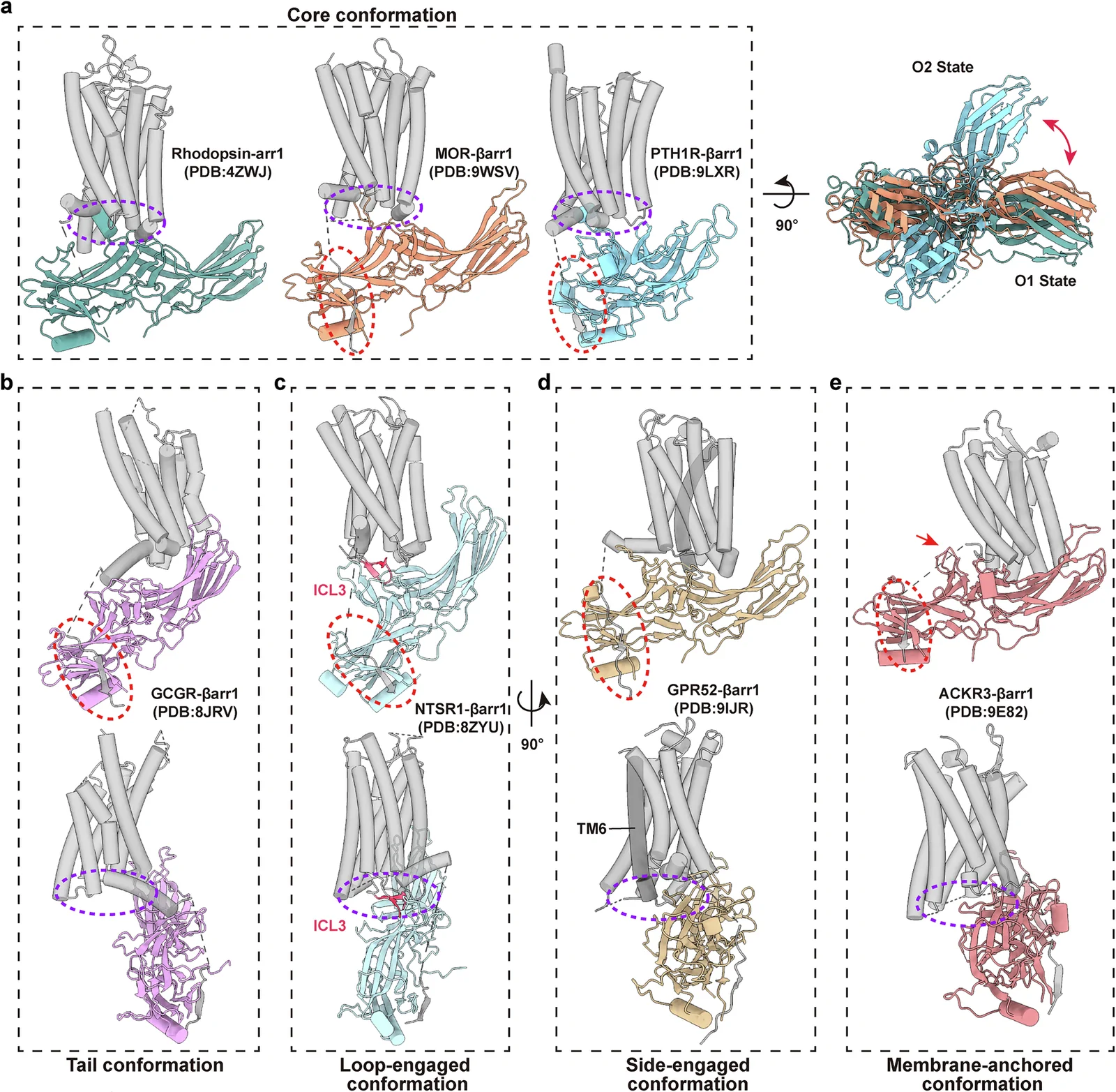
The structural continuum of GPCR–arrestin engagement conformations. **a** Canonical core-engaged conformation. Representative structures of the core conformation are shown, including rhodopsin–arrestin-1 (PDB: 4ZWJ), MOR–βarr1 (PDB: 9WSV), and PTH1R–βarr1 (PDB: 9LXR) complexes. The receptor TMD core and C-terminal tail are highlighted by purple and red circles, respectively. Comparisons of intracellular arrestin orientations are shown on the right; the canonical state (O1, observed in rhodopsin) and the rotated state (O2) illustrate conformational plasticity within the core-engaged mode. **b** Tail-engaged conformation. The tail-engaged conformation is exemplified by the GCGR–βarr1 complex (PDB: 8JRV). Two perspectives highlight the exclusive tethering via the C-tail (top panel, red circle) and the unoccupied, sterically accessible TMD core (bottom panel, purple circle), which permits simultaneous G protein coupling. **c** Loop-engaged conformation. The loop-engaged conformation was captured in the NTSR1–βarr1–SBI-553 complex (PDB: 8ZYU). The remodeled ICL3 of NTSR1 (colored red and highlighted) inserts into the arrestin central crest, while the arrestin finger loop remains external to the receptor core. **d** Side-engaged conformation. The noncanonical side-engaged mode observed in the GPR52–βarr1 complex (PDB: 9IJR). Arrestin binds laterally via ICL1 and helix 8 without penetrating the receptor core. TM6 remains in an inactive-like conformation, supporting constitutive, agonist-independent arrestin recruitment. **e** Membrane-anchored conformation. The ACKR3–βarr1 complex (PDB: 9E82) is characterized by a membrane-anchored arrestin conformation. The arrestin finger loop (indicated by a red arrow) inserts directly into the membrane/micelle boundary rather than the receptor TMD core. Note that the TMD core (purple circle) remains unoccupied, while the complex is tethered by the C-tail (red circle).

The static duality of core- vs tail-engaged conformations has been superseded by the dynamic engagement continuum, where arrestin samples a landscape of metastable intermediates that tune the signaling output. Canonical core-engaged conformations (e.g., rhodopsin^15,21,22^, μ-opioid receptor (MOR)^39^, and PTH1R (PDB: 9LXR)^40^) involve deep finger-loop insertion to sterically block G proteins and drive desensitization (Fig. 2a), whereas the tail-engaged conformation (e.g., GCGR^29^) tethers β-arrestin primarily via the phosphorylated C-terminus and permits megacomplex formation and sustained signaling^42^ (Fig. 2b).

The loop-engaged conformation, which was first structurally defined in the neurotensin receptor 1 (NTSR1)–βarr1–SBI-553 complex^33^, represents a β-arrestin-selective receptor conformation (Fig. 2c, SBI-553 is a β-arrestin-biased allosteric modulator discussed in the section of “Structural mechanisms of biased signaling”). In this state, the βarr1 finger loop remains outside the transmembrane cavity, whereas the remodeled ICL3 loop of NTSR1 inserts into the central crest of βarr1 in a “hooking” manner. This architecture sterically excludes G proteins but preserves arrestin scaffolding, explaining modulator-driven β-arrestin bias.

The side-engaged conformation is a distinct noncanonical mode observed in the orphan receptor G protein-coupled receptor 52 (GPR52)^35^ (Fig. 2d). βarr1 binds laterally via ICL1 and helix 8 of GPR52, without core penetration or the outward movement of TM6. The receptor remains in an inactive-like conformation stabilized by a conserved D^3.49^-R^3.50^-I^5.62^-A^6.34^ motif, enabling constitutive, agonist-independent βarr1 recruitment^35^. This mode functions as a regulatory mechanism rather than an activity-dependent switch.

The membrane-anchored conformation, as observed in the ACKR3–βarr1 complex^34^, represents a unique variant of tail-type binding (Fig. 2e). Here, βarr1 is tethered to the phosphorylated C-terminus, yet its finger loop, which is rich in conserved hydrophobic residues, inserts into the lipid/micelle boundary rather than the receptor core^34^. Functionally, this lipid anchoring (involving both the finger loop and C-edge) promotes rapid capture dictated by the phosphorylation barcode, but importantly does not occlude the cytoplasmic cleft. This interaction leaves the TMD accessible for G protein or GRK access, establishing a membrane-coupled stabilized state that facilitates recruitment without enforcing canonical desensitization.

Together, these structures establish that GPCR–arrestin coupling spans core, loop, side, membrane-anchored, and tail conformations, forming a continuous structural spectrum that decouples arrestin recruitment from receptor activation and provides a mechanistic framework for signaling bias.

## The logic of assembly: multi-dimensional modulation

Arrestin is not a passive adapter but a conditional signal integrator that decodes layered regulatory inputs into distinct functional outputs. The assembly of GPCR–arrestin complexes is specified by the coordinated actions of the GRK-written phosphorylation barcodes, lipid cofactors, and isoform-dependent structural features that bias membrane engagement and conformational stability, thereby shaping the arrestin microstate landscape.

### The expanded phosphorylation barcode

The recruitment and activation of arrestin are governed by a sophisticated “phosphorylation barcode”^17–20^, a concept established by seminal work on the β2-adrenergic receptor (β_2_AR) and M3 muscarinic receptor (M3R). These studies demonstrated that distinct spatial phosphorylation patterns that are actively written by specific GRK isoforms function dually as a recruitment dock and an allosteric activation trigger. As described in the section of “Capture of GPCR–arrestin complexes”, multivalent phosphate engagement disrupts the polar core and three-element autoinhibitory interactions^48,49^, triggering the conformational switch required for productive receptor engagement^16,45,47^ (Fig. 1b, c). However, arrestin activation is not uniform; instead, the specific pattern and location of phosphorylation determine which functional conformation is stabilized. This pattern-dependent activation is validated by mass spectrometry-based phosphoproteomics, mutagenesis, and real-time BRET biosensors^75–78^.

**Fig. 3:**
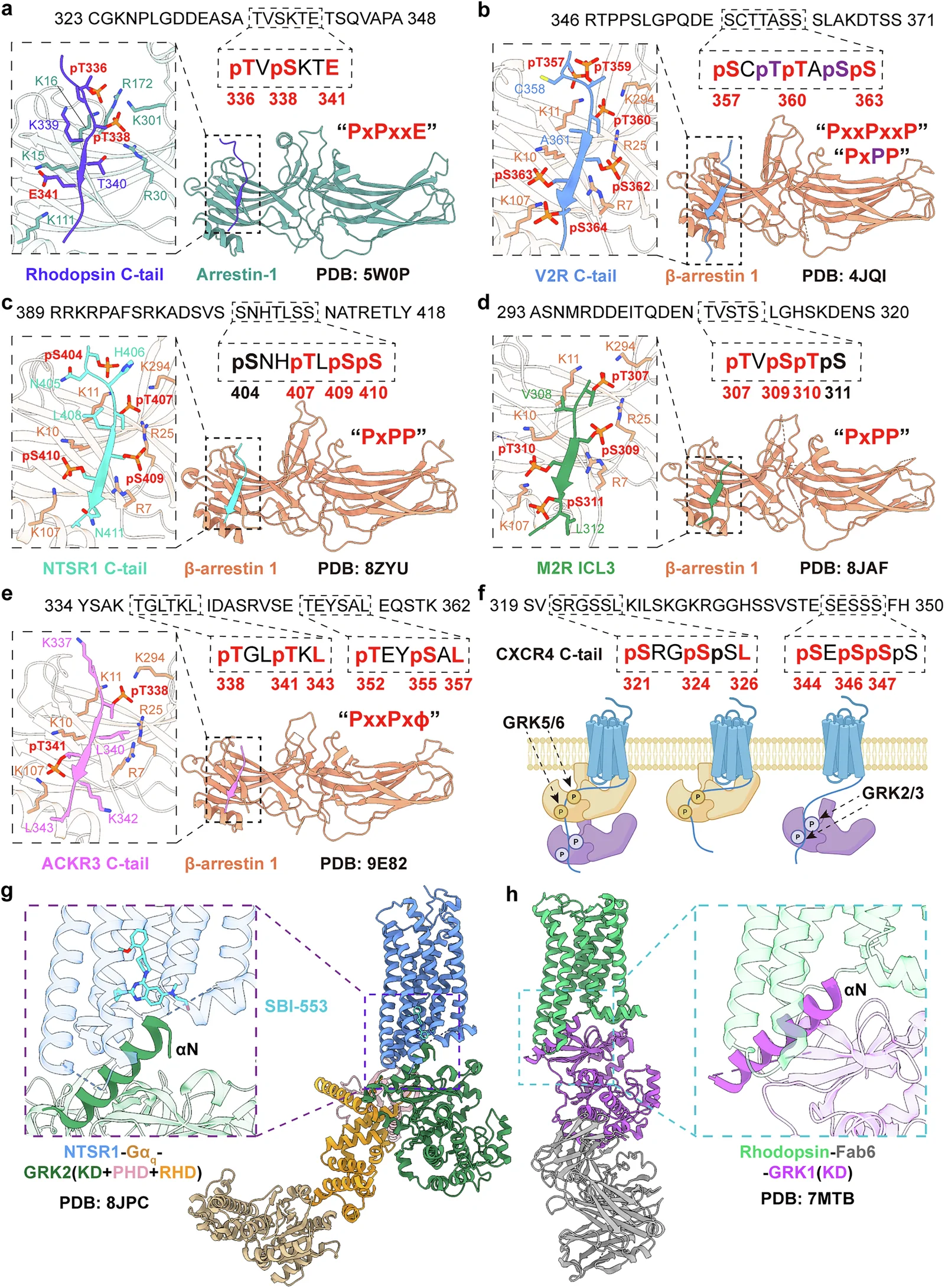
Diversity of phosphorylation barcodes in the GPCR C-terminus and ICL3. **a** Classical C-terminal phosphorylation barcode “PxPxxP/E/D” in rhodopsin, as observed in the rhodopsin–arrestin-1 complex (PDB: 5W0P). **b** Classical C-terminal phosphorylation barcode “PxxPxxP/E/D” in V2R, as resolved in the V2Rpp–βarr1 complex (PDB: 4JQI). **c**, **d** The “PxPP” phosphorylation barcode located in the C-terminus of NTSR1 in the NTSR1‒βarr1 complex (PDB: 8ZYU) (**c**) and in the ICL3 of M2R in the M2Rpp‒βarr1 complex (PDB: 8JAF) (**d**). **e** Distinct “PxxPxϕ” phosphorylation barcodes in the proximal and distal C-tail of ACKR3 in the ACKR3–βarr1 complex (PDB: 9E82). The proximal barcode is preferentially phosphorylated by GRK5, whereas the distal barcode is selectively modified by GRK2. **f** Schematic illustration of proximal and distal phosphorylation barcodes in ACKR3 and CXCR4, highlighting differential GRK specificity and spatial organization (the figure was created with BioRender). **g** Structure of the NTSR1‒GRK2‒Gα_q_ complex (PDB: 8JPC) showing the insertion of the N-terminal helix (αN) of GRK2 into the TMD core of NTSR1. KD, kinase domain; PHD, pleckstrin homology domain; RHD, regulatory G protein signaling homology domain. **h** Structure of the rhodopsin‒GRK1‒Fab6 complex (PDB: 7MTB) illustrating the insertion of the αN of GRK1 into the TMD core of rhodopsin.

Zhou et al. fundamentally demonstrated that specific spatial arrangements of phosphates, rather than total negative charge, determine the fidelity of arrestin recruitment and activation through the X-ray structure^21^. Based on the crystal structure of the rhodopsin‒arrestin-1 complex (PDB: 5W0P) and extensive sequence analysis, they identified a recurrent consensus phosphorylation code, Px(x)PxxP/E/D, where “P” denotes a phosphorylated serine or threonine. In this mechanism, the precise spacing of phosphorylated residues or acidic amino acids (aspartate or glutamate) is positioned to engage discrete positively charged phosphate-sensor pockets in the arrestin-1 N-domain (including K15, K16, R30, K111, R172, and K301), displacing the arrestin-1 C-tail and initiating activation^21^ (Fig. 3a). In support of this phosphorylation barcode, the V2R C-terminus contains an extended PxxPxxP motif that tightly engages the βarr1 N-domain^16^ (Fig. 3b).

Full Px(x)PxxP/E/D motifs maximize binding affinity through multivalent electrostatic networks with the arrestin N-domain^79–81^. Structural and biochemical analyses of diverse GPCRs, including C-X-C chemokine receptor type 7 (CXCR7), have demonstrated that these canonical clusters coordinate tightly with the basic pockets of arrestin to dictate the conformation of the engaged receptor C-tail^80,81^. However, while extended tracks drive high-affinity assembly, they are not strictly required to encode specific functional outcomes. Indeed, even a single key phosphorylation site can act as a crucial structural anchor to direct interdomain rotation and regulate trafficking^82^. Recognizing this functional diversity, the structural focus of the field has naturally expanded to investigate how alternative phosphorylation barcodes also efficiently coordinate the activation of the complexes.

Consequently, structural studies further refined the phosphorylation barcode concept. Structural analyses of β-arrestin complexes with C-C chemokine receptor type 5 (CCR5) and other GPCR phosphopeptides revealed that a PxPP/E/D motif^45,47,83^ in which two phosphorylated residues are separated by a single amino acid and followed by an additional phosphate or acidic residue acts as a privileged activating geometry when it is present in GPCR phosphotails (such as NTSR1^33^) or ICL3 (such as M2 muscarinic receptor (M2R)^45,47^) (Fig. 3c, d). Structural snapshots of β-arrestin bound to diverse GPCR-derived phosphopeptides show that this PxPP pattern engages a conserved “K-K-R-R-K-K lock” (a network of positively charged residues) within the arrestin N-domain, promoting the displacement of the arrestin C-terminus^45,47^. However, the PxPP sequence is sufficient but not essential. Many GPCRs, including several atypical chemokine receptors (e.g., ACKR3^34^), lack the PxPP sequence yet still recruit β-arrestin via alternative phosphorylation patterns that stabilize distinct active conformations. Thus, PxPP represents a high-efficiency barcode element embedded within a broader, combinatorial phosphorylation code in which the motif composition, spacing, and GRK-specific installation collectively tune the arrestin conformation and downstream signaling.

Recent structural insights have expanded the classical phosphorylation code to include an alternative “hydrophobic anchor” motif that was identified in ACKR3 as x(pT)xx(pS/T)xϕ, where ϕ represents a medium-sized hydrophobic residue^34^ (Fig. 3e, f). In this model, β-arrestin activation is facilitated by the terminal hydrophobic residue docking into a complementary hydrophobic pocket on the arrestin surface, providing a critical anchoring point that stabilizes the active complex. A bioinformatic analysis revealed that while the minimal PxxP core is ubiquitous (~91% of class A GPCR C-tails, as recently revealed in GPR1 for the PxxE motif^38^), high-affinity β-arrestin engagement typically requires an additional “plus-one” element: either a third phosphate, as in the PxPP motif (~68%), or a hydrophobic anchor, as in PxxPxϕ (~63%)^34^. This expanded barcode repertoire allows arrestin to decode a broader spectrum of receptors using distinct chemical and stereochemical cues.

The functional logic of these barcodes was illuminated by the mechanistic studies of Latorraca et al.^84^; by detailing the molecular effects of phosphorylation barcodes on βarr1, their work has key implications for arrestin function. Specifically, they demonstrated that arrestin binding and activation are separable processes encoded by distinct phosphorylation patterns. Some barcodes primarily act as binding codes, stabilizing arrestin at the receptor, whereas activation codes are required to drive interdomain rotation, finger-loop engagement, and other conformational changes required for arrestin-mediated signaling^84^. Consequently, strong binding does not necessarily equate to functional activation: a receptor may recruit β-arrestin without inducing downstream signaling or conversely induce partial or transient activation with limited binding, which is consistent with the catalytic activation characteristic of β-arrestin^85^. Importantly, barcode function depends less on the total number of phosphates than on their spatial arrangement and context, as individual sites can exert stimulatory or inhibitory effects depending on their combinatorial placement. Thus, GPCR phosphorylation operates as a combinatorial logic gate, independently tuning arrestin recruitment, conformational activation, and effector coupling.

### The modulation of GRKs: topological writers of the barcode

Phosphorylation barcodes are not intrinsic properties of GPCRs alone but are actively written by distinct GRKs^19,86^. GRK isoform specificity determines the location, composition, and kinetics of receptor phosphorylation, thereby shaping arrestin recruitment, activation, and downstream functional outcomes. Accumulating biochemical, structural, and cellular evidence indicates that much of what is described as “arrestin bias” is, in practice, GRK-dependent barcode bias^87–89^. Structural work has shown how this process occurs in atomic detail: the structure of rhodopsin–GRK1^90^ first established the mode of GRK engagement, and more recent structures reveal GRK2 assembling onto NTSR1^91^, with its N-terminal helix inserting into the intracellular cavity to position the kinase domain for access to cytoplasmic phosphorylation sites — a process that is further promoted by the intracellular biased modulator SBI-553, demonstrating how ligands and modulators tune GRK engagement and thereby encode the phosphorylation barcode^90,91^ (Fig. 3g, h).

Functionally, GRK2/3 are largely cytosolic and are recruited to activated receptors by Gβγ (and other adapters), which biases them toward phosphorylating more distal, high-density clusters on C-tails — a pattern associated with rapid β-arrestin binding and dynamic, internalization-prone complexes^19,34,92,93^. In contrast, GRK5/6 is more strongly associated with the membrane and preferentially phosphorylates proximal, site-specific motifs near the membrane, producing barcodes that stabilize long-lived, scaffolding-competent β-arrestin states^19,34,92,93^. These mechanistic contrasts underlie “barcode bias”: the same receptor can produce qualitatively different arrestin outcomes depending on which GRKs dominate. Furthermore, the use of a barcode system depends on the cellular context, as illustrated for ACKR3. Because it lacks canonical G protein coupling, ACKR3 cannot independently liberate Gβγ and thus relies primarily on GRK5-mediated phosphorylation^92^. However, it can dynamically recruit GRK2 through the release of Gβγ subunits during the co-activation of CXCR4^34,92^. Through these mechanisms, the phosphorylation barcode extends beyond isolated intra-receptor regulation and instead functions as a sophisticated sensor of inter-receptor crosstalk and the broader cellular environment.

Recent receptor-specific structures of ACKR3^34^ and CXCR4^45,93^ have provided structural proof of this principle (Fig. 3e, f). A comparative analysis of ACKR3 complexes revealed that the specific location of the phosphorylation barcode dictates the β-arrestin binding mode: proximal phosphorylation by GRK5 promotes robust C-edge membrane anchoring and stable finger-loop contacts with the lipid/micelle surface (PDB: 9E82), whereas GRK2-mediated phosphorylation of the distal C-tail yields a more transient interaction with minimal membrane penetration (e.g., PDB: 8TII)^34^. These distinct phospho-engagements alter the orientation of the finger loop of β-arrestin relative to that of the lipid micelle and modulate the stability of membrane anchoring. This mechanism directly links GRK identity to arrestin geometry and dwell time, determining the balance between stable scaffolding and transient trafficking. Hence, controlling GRK localization or activity is important for tuning arrestin-dependent signaling.

### Lipid cofactors as allosteric regulators

While receptor phosphorylation patterns provide sequence-encoded signals for arrestin recruitment, its stabilization and activation are further modulated by a “lipid code” comprising membrane-anchored phosphoinositides and soluble inositol phosphates^50,51,94–96^. Together, these factors constitute a “lipid code” that functions not only as an anchor but also as an allosteric regulator that defines receptor class phenotypes and temporal signaling dynamics.

PIP_2_, a membrane-bound lipid, plays a central role in regulating β-arrestin by binding basic patches on the arrestin C-lobe. Functional and structural analyses have demonstrated that PIP_2_ acts as an allosteric cofactor that stabilizes an active-like β-arrestin intermediate, increasing finger loop and gate loop dynamics and increasing reactivity toward active-state sensors such as Fab30, even in the absence of a GPCR^50,94,95^. By binding to the C-lobe basic patch, plasma membrane (PM) phosphoinositides (such as PI(4,5)P_2_ and PI(3,4,5)P_3_) — but notably not the lipid PI(3)P — lower the energetic barrier for GPCR core engagement and effectively shift the equilibrium toward an active state without requiring full C-tail displacement^50^. This lipid-dependent activation provides a molecular explanation for the distinction between class A and class B GPCRs, which are classified by their arrestin binding affinities and the duration of receptor–arrestin interactions^97^. Class A receptors typically require the coincident detection of phosphorylation and PIP_2_ for robust recruitment (confining activity to PIP_2_-rich PM domains), whereas class B receptors with strong phosphorylation barcodes can bypass the PIP_2_ requirement and sustain β-arrestin association in endosomes^50^.

In addition to allosteric priming, Kuramoto et al. recently revealed that this regulation relies on a multivalent interaction; they identified a noncanonical PIP_2_ binding site at the tips of the β-arrestin C-domain that works synergistically with the known canonical site^98^. This multivalent binding precedes stable C-edge loop insertion into the lipid bilayer^98^. Together, these sequential processes ensure the formation of stable GPCR–β-arrestin complexes, driving their rapid accumulation into specialized membrane domains to promote efficient receptor desensitization and endocytosis.

**Fig. 4:**
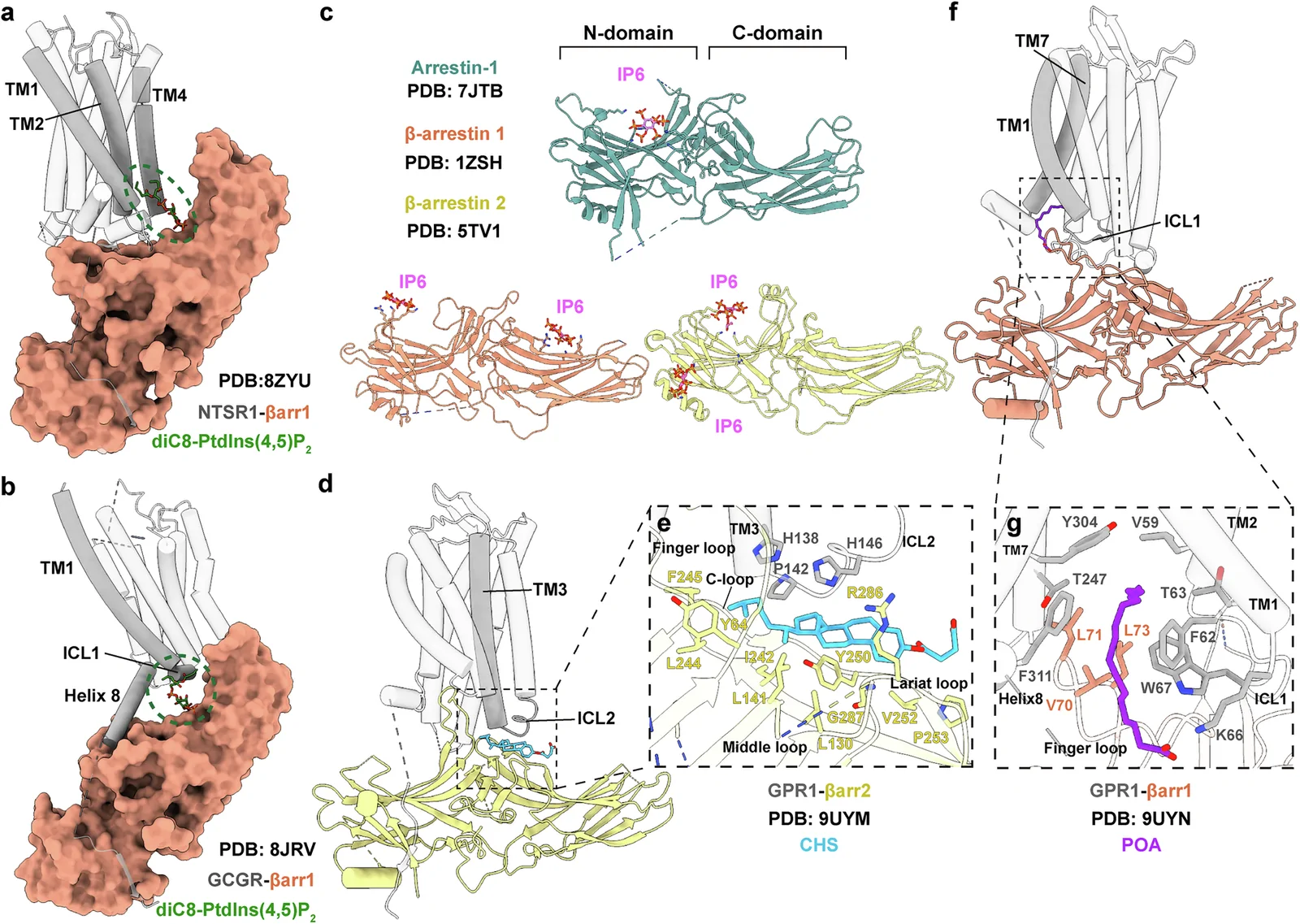
Lipids as modulators of the arrestin structure and GPCR engagement. **a**, **b** Binding modes of the phosphoinositide diC8-PtdIns(4,5)P_2_ within the C-edge of arrestin in the NTSR1–βarr1 complex (PDB: 8ZYU) (**a**) and GCGR–βarr1 complex (PDB: 8JRV) (**b**). **c** Comparative analysis of IP6 binding sites across the arrestin-1 (PDB: 7JTB), βarr1 (PDB: 1ZSH), and βarr2 (PDB: 5TV1) isoforms, highlighting the conserved polar pocket. **d**, **e** Structural characterization of the CHS binding site in the GPR1–βarr2 complex (PDB: 9UYM), showing an overall view of the lipid‒protein interface (**d**) and a detailed representation of the specific coordinating residues (**e**). **f**, **g** Structural characterization of the POA binding site in the GPR1–βarr1 complex (PDB: 9UYN), illustrating the global orientation of the fatty acid (**f**) and a close-up view of the hydrophobic interactions (**g**).

The stabilization of β-arrestin by PIP_2_ was verified by the GPCR–β-arrestin structures, such as NTSR1‒βarr1^24,33^ and GCGR‒βarr1^29^ (Fig. 4a, b). In the NTSR1–βarr1 complex, diC8-PtdIns(4,5)P_2_ acts as a molecular bridge: its 4,5-bisphosphate headgroup engages basic residues on the C-lobe of βarr1, while its lipid tail packs against the membrane-facing surfaces of TM1, TM2, and TM4 of NTSR1^24,33^. In the GCGR–βarr1 complex, diC8-PtdIns(4,5)P_2_ adopts a related binding mode, with 4,5-bisphosphate similarly coordinating the βarr1 C-lobe, while an additional bridging phosphate interacts with residues in TM1, ICL1, and helix 8 of the GCGR, thereby reinforcing receptor–βarr1 coupling^29^.

While soluble diC8-PI(4,5)P_2_ provides vital structural insights, it inherently lacks the extended planar surface of a physiological lipid bilayer. This limitation prevents the simultaneous engagement of multiple membrane-binding elements distributed across both the N-domain and the C-domain of arrestin. Furthermore, highly curved environments (such as small-diameter nanodiscs) cause the arrestin C-edge in GPCR–β-arrestin complexes to “hang” without a stable membrane contact and destabilize the active complex^25^. To overcome these limitations, recent biophysical studies utilizing extended flat lipid nanodiscs have revealed that the two β-arrestins deploy distinct membrane-anchoring mechanisms^99^. Basal βarr1 selectively targets PI(4,5)P_2_-enriched nanodiscs via its C-edge with high affinity, shifting to finger-loop engagement upon activation to support further stabilization. In contrast, βarr2 binds more broadly to various anionic lipids (PI(4,5)P_2_, POPS, and POPG)^99^. Although the lipid bilayer alone is insufficient to fully activate either isoform, it synergistically increases the recruitment and activation of both β-arrestins in the presence of a phosphorylated GPCR C-tail. Ultimately, the lipid environment serves as a crucial, isoform-specific modulator that finely tunes arrestin recruitment and activation dynamics.

In addition to membrane lipids, soluble IP6 functions as an isoform-specific allosteric regulator that differentially modulates arrestin conformational equilibria and oligomeric states. In βarr2, IP6 functions as a potent structural activator; by targeting the N-domain phosphate sensor to mimic a phosphorylated receptor C-tail, it disrupts the central polar core and induces the interdomain twist to stabilize active trimers^52^ (Fig. 4c). In contrast, IP6 serves primarily as a conformational priming agent for arrestin-1. Here, IP6 binding displaces the autoinhibitory C-tail but leaves the polar core intact, dissociating basal oligomers into functional monomers without inducing active twisting^55,100,101^ (Fig. 4c). Finally, IP6 serves as a negative regulator of βarr1. Through a unique two-site binding mechanism, IP6 drives the formation of inactive cytosolic βarr1 oligomers^101,102^ (Fig. 4c). By promoting this oligomeric state, IP6 effectively sequesters the protein in the cytoplasm, preventing free βarr1 monomers from translocating to the nucleus or interacting with PM receptors^102^.

Finally, specific interfacial sterols and lipids can act as structural cofactors to tune isoform selectivity and constitutive activity^38^. Recent cryo-EM analyses of the GPR1‒βarr2 complex revealed that cholesterol wedges between ICL2 of the receptor and the central loops of βarr2, strengthening receptor‒βarr2 binding via extensive hydrophobic contacts^38^ (Figs. 1f and 4d, e). Furthermore, the endogenous fatty acid palmitoleic acid (POA) has been shown to be involved in constitutive recruitment. Structural data revealed that binding of the POA to GPR1 enhances agonist-independent capture of βarr1^38^ (Fig. 4f, g).

## Structural mechanisms of biased signaling

The G protein and arrestin pathways constitute two fundamentally different modes through which GPCRs transduce extracellular information^3^. G proteins rapidly relay signals to classical second-messenger cascades, resulting in biochemical responses^103,104^; arrestins then lead to the desensitization of G protein signaling and the internalization of the receptors and simultaneously act as scaffolds for distinct downstream modules^7–14,105,106^. Mechanistically, the receptor-induced conformational activation of β-arrestin exposes binding interfaces that scaffold downstream kinase cascades^5,13,107,108^. This structural rearrangement enables the physical engagement of SH3 domains within Src family kinases and the coordinated activation of mitogen-activated protein kinase (MAPK) pathways, such as the c-Raf-MEK1-ERK1/2 axis and the JNK cascade^11,108–115^. By spatially restricting these signaling complexes, β-arrestin translates receptor engagement into precise, G protein-independent physiological responses^11,107,108,116^.

**Fig. 5:**
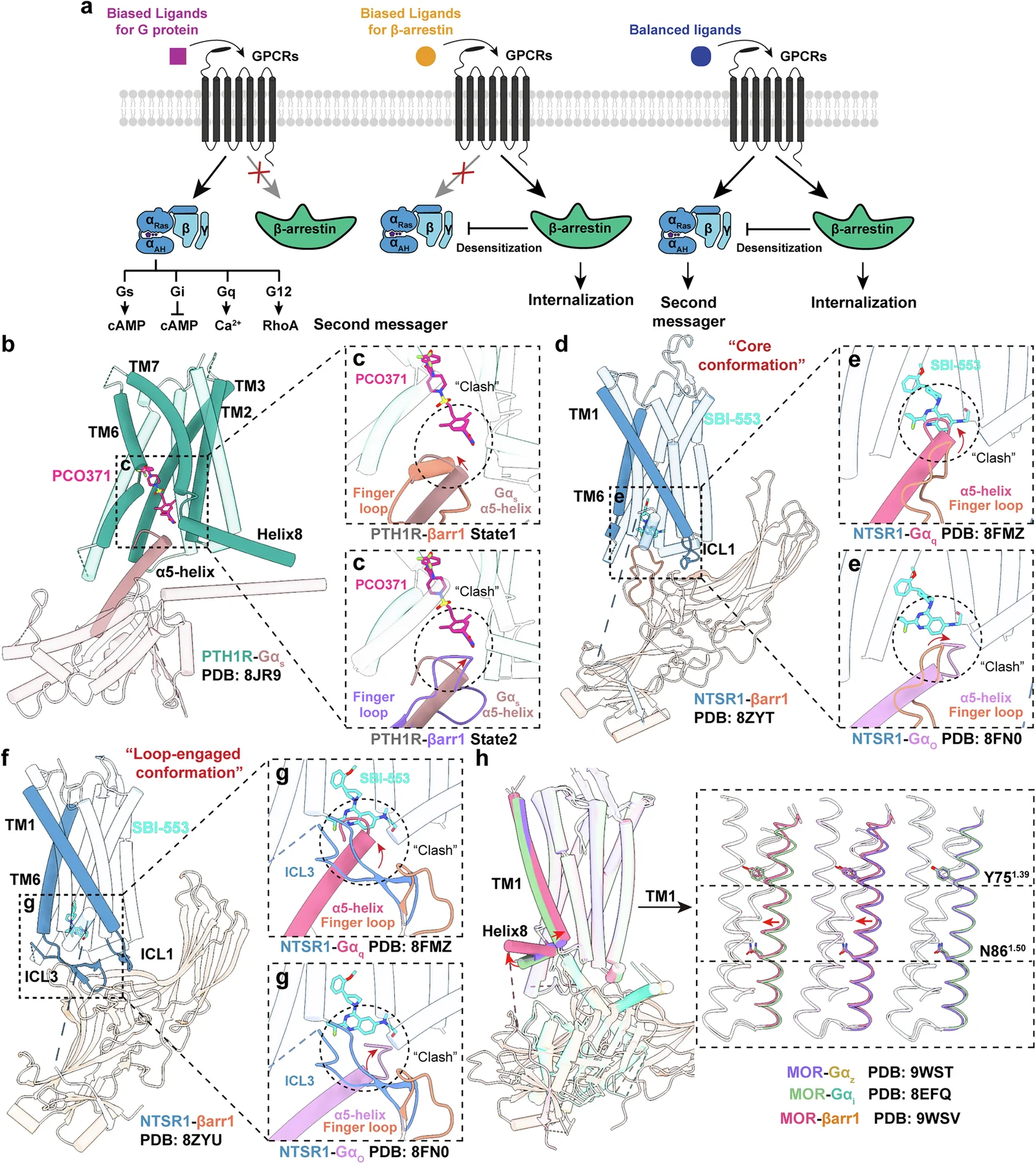
Structural basis of biased GPCR signaling toward G proteins or β-arrestins. **a** Conceptual schematic illustrating ligand-directed signaling bias, in which GPCR ligands preferentially stabilize G protein-biased, β-arrestin-biased, or balanced signaling states, leading to distinct downstream signaling outputs. **b** Illustration of the G_s_-biased conformation stabilized by the ligand PCO371 in the PTH1R‒G_s_ complex (PDB: 8JR9). **c** Detailed comparison of the G_s_-bound state and βarr1-bound states of PTH1R, highlighting steric clashes between PCO371 and the βarr1 finger loop in both State 1 (top panel; PDB: 9LXR) and State 2 (bottom panel; PDB: 9LXP). **d** The canonical core-engaged conformation of the NTSR1–βarr1 complex stabilized by the biased allosteric modulator SBI-553 (PDB: 8ZYT). **e** Structural alignments of the SBI-553-bound NTSR1–βarr1 complex (PDB: 8ZYT) with the canonical NTSR1–G_q_ (top panel; PDB: 8FMZ) and NTSR1–G_o_ (bottom panel; PDB: 8FN0) complexes. This superposition shows that the binding position of SBI-553 sterically clashes with the incoming Gα α5-helix, effectively blocking G protein coupling while seamlessly accommodating the βarr1 finger loop. **f** The loop-engaged conformation of the NTSR1–βarr1 complex stabilized by SBI-553 (PDB: 8ZYU). In this unique state, the arrestin finger loop is entirely excluded from the receptor core; instead, ICL3 of the receptor docks directly into the cavity of the βarr1 central crest. **g** Comparison of the loop-engaged NTSR1–βarr1 complex (PDB: 8ZYU) with the canonical NTSR1–G_q_ (top panel) and NTSR1–G_o_ (bottom panel) complexes. The superposition illustrates that SBI-553 occupies the intracellular cavity to sterically occlude G protein coupling, forcing arrestin to engage via a distinct interaction interface mediated by ICL3 of the receptor. **h** Structural role of TM1 in determining signaling specificity for MOR. Superposition and comparison of the MOR‒G_z_ (PDB: 9WST), MOR‒G_i_ (PDB: 8EFQ), and MOR‒βarr1 (PDB: 9WSV) complexes highlight the conformational differences in TM1.

The functional divergence of these two signaling branches — G protein-driven second messengers vs β-arrestin-mediated desensitization and kinase scaffolding — provides the biological foundation for biased agonism^57,117^. The concept of biased signaling captures the observation that chemically distinct ligands acting at the same receptor can differentially engage these outputs, namely, the ability of specific ligands to selectively stabilize receptor conformations that activate one cytosolic transducer (e.g., G protein or β-arrestin) while silencing the other^58,118–121^ (Fig. 5a). This paradigm shift offers a route to dissociate therapeutic efficacy from on-target side effects, a concept that has now been validated by high-resolution structural biology.

The first major structural breakthrough in defining the atomic basis of bias arose from the study of class B1 GPCRs, specifically PTH1R bound to the small molecule PCO371 and the G_s_ protein^122,123^. Unlike endogenous peptide agonists that bind the extracellular domain, PCO371 engages a conserved intracellular pocket formed by TM2, TM3, TM6, TM7, and helix 8^122^ (Fig. 5b). This intracellular binding site acts as an allosteric wedge. By occupying the G protein coupling cavity directly, PCO371 stabilizes a distinct outward rotation of TM6 that is optimal for G_s_ activation but sterically incompatible with the broad interdomain interface required for β-arrestin recruitment (Fig. 5c). This finding proves that bias can be engineered by targeting conserved intracellular cavities to physically enforce a specific transducer-coupling geometry.

With advancements in complex stabilization, the field subsequently resolved the mechanism of β-arrestin bias. The landmark structure of the NTSR1 complexed with the IAM SBI-553 provided the definitive mechanism for β-arrestin bias^33,91^. Mechanistically, SBI-553 achieves profound bias by occupying the intracellular transmembrane cavity to sterically occlude G protein coupling while simultaneously accommodating βarr1 recruitment through two distinct spatial modes^33,124^ (Fig. 5d–g). In the canonical core-engaged conformation, SBI-553 allows the βarr1 finger loop to seamlessly co-occupy the receptor pocket (Fig. 5d, e). Strikingly, SBI-553 also stabilizes an unprecedented loop-engaged conformation. In this state, the βarr1 finger loop does not insert into the receptor core; instead, ICL3 of the receptor docks directly into the cavity of the βarr1 central crest (Fig. 5f, g). This interface is created by significant conformational remodeling of the ICL3–TM6 and TM1–ICL1 regions, which form extensive new contacts with the βarr1 loops. Notably, a structural comparison with canonical complexes^23,24^ suggests that this loop engagement is not a naturally occurring intermediate but a synthetic state induced specifically by a biased allosteric modulator to bypass the steric constraints of the drug-occupied core.

Most recently, structural efforts have redefined the understanding of orthosteric bias by mapping the complete effector landscape of the MOR^39^. Structures of the MOR complexed with its distinct transducers — G_i_^125^, G_z_, and βarr1^39^ — reveal that signaling selectivity arises from conformational preferences encoded outside the canonical intracellular coupling pocket. Specifically, TM1 has been identified as a previously underappreciated allosteric regulator of this bias (Fig. 5h). A structural analysis demonstrated that TM1 acts as a transmission gear for transducer selectivity through distinct conformational dynamics: outward TM1 movement promotes the structural flexibility necessary for G protein coupling, whereas inward TM1 rearrangement stabilizes the intracellular binding pocket to facilitate βarr1 recruitment^39^. Residues within the newly identified “TM1 fusion pocket” (specifically Y75^1.39^ and N86^1.50^) modulate ligand binding and induce conformational fluctuations that propagate to the intracellular TM2–TM7–helix 8 interface, thereby differentially modulating the coupling efficiency of the G protein vs β-arrestin^39^. This mechanism establishes TM1 not as a static scaffold but as a dynamic allosteric switch driven by thermodynamic heterogeneity, potentially linking biased signaling to oligomerization interfaces where TM1 frequently governs receptor assembly.

## Noncanonical endosomal signaling and megacomplex formation

### Noncanonical endosomal signaling

**Fig. 6:**
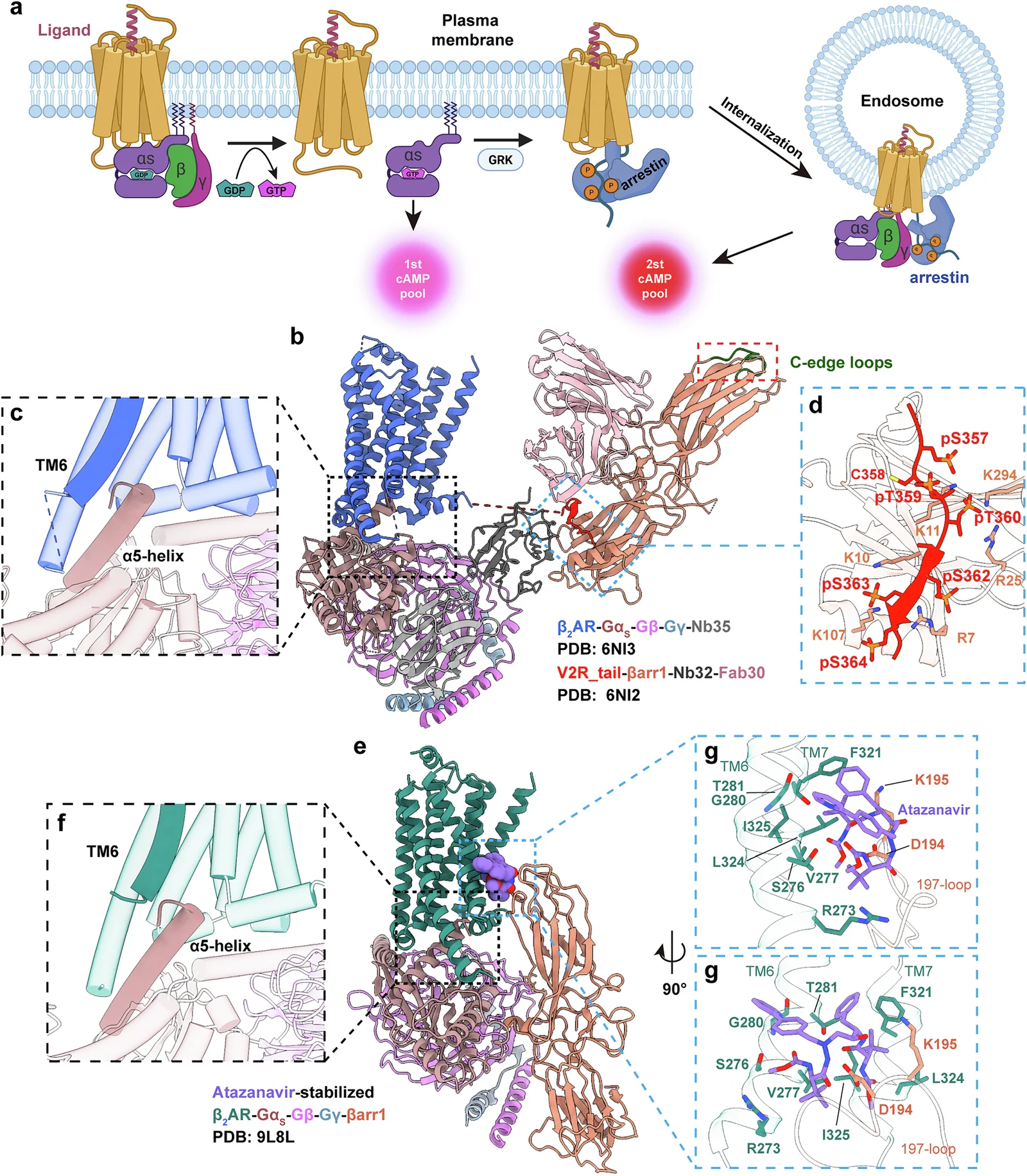
Endosomal GPCR signaling and the GPCR–G protein–β-arrestin megacomplex. **a** Schematic illustration of biphasic cAMP signaling, consisting of a rapid, transient phase initiated at the plasma membrane followed by a sustained signaling phase originating from endosomes after receptor internalization (created with BioRender). **b** Overall architecture of the β2V2R‒G_s_‒βarr1 megacomplex. The composite model is assembled from the β_2_AR‒G_s_‒Nb35 (PDB: 6NI3) and V2R_tail‒βarr1‒Nb32‒Fab30 (PDB: 6NI2) complexes, illustrating the simultaneous engagement of the G protein and arrestin by a single receptor. **c** Close-up view of the Gα_s_ α5-helix inserted into the TMD of β2V2R, highlighting canonical G protein coupling within the megacomplex configuration. **d** Detailed view of the interaction of the phosphorylated V2R C-tail with βarr1, demonstrating tail-mediated arrestin engagement that coexists with active G protein coupling and underlies sustained endosomal signaling. **e** Overall architecture of the atazanavir-stabilized β_2_AR_3muts‒G_s_‒βarr1 megacomplex (PDB: 9L8L), demonstrating that simultaneous coupling is facilitated by the small molecule atazanavir. **f** Close-up of canonical Gα_s_ α5-helix engagement within the β_2_AR core. **g** Detailed view of the atazanavir-mediated interface between β_2_AR_3muts and βarr1.

For decades, the “canonical” model suggested that GPCR signaling is strictly confined to the PM, with arrestin recruitment functioning primarily as a termination mechanism that uncouples G proteins and drives internalization. This paradigm was revised by the discovery of endosomal signaling, which was first visualized in the pioneering works of Calebiro et al.^63^ and Ferrandon et al.^126^, who utilized Förster resonance energy transfer (FRET)-based biosensors to demonstrate persistent cAMP generation from internalized thyroid-stimulating hormone receptor and PTH1R. Endosomes serve as discrete signaling compartments that generate “cAMP waves” distinct from those generated by PM signaling (Fig. 6a). Endosomal signaling is now recognized as a general feature of multiple GPCRs, including β_2_AR^127^ and V2R^128^, with important implications for signal duration, location, and specificity^129–131^. Spatial encoding enables cells to distinguish transient PM-derived signals from sustained endosomal signaling programs, thereby selectively engaging gene expression pathways that are inaccessible to acute PM signaling^132,133^.

Among these receptors, PTH1R represents the most mechanistically complete model of endosomal signaling, integrating the ligand residence time, β-arrestin scaffolding, G protein coupling, and spatial signal termination^134–139^. PTH1R signaling proceeds through a biphasic cAMP program: a rapid PM phase followed by a sustained endosomal phase that is essential for downstream transcriptional and physiological responses^134–136^.

Following ligand binding at the PM, PTH1R activates G_s_, inducing an acute increase in cAMP levels. Almost immediately, GRK-mediated phosphorylation of the receptor promotes β-arrestin recruitment and internalization while stabilizing a signaling-competent receptor state rather than enforcing immediate desensitization. Crucially, a long ligand residence time allows PTH1R to remain active during endocytosis, sustaining cAMP production from early endosomes^126^. Within endosomes, β-arrestin not only coexists with G protein but also actively scaffolds signaling complexes that support continued cAMP production^135,140^. In particular, β-arrestin-bound PTH1R can engage Gβγ subunits, forming a PTH1R–β-arrestin–Gβγ signaling node that contributes to downstream signaling and reinforces sustained cAMP generation^140^. This β-arrestin-mediated organization explains how endosomal PTH1R signaling remains robust even when PM G protein activation has terminated.

While sustained endosomal signaling is critical for physiological responses, cells employ multiple mechanisms to actively terminate this signaling and prevent pathological overactivation. Local cAMP activates PKA, which phosphorylates signaling components to decrease receptor activity, whereas v-ATPase-driven endosomal acidification gradually destabilizes ligand–receptor interactions^138^. Finally, signal termination is executed by the retromer complex, which selectively sorts PTH1R from signaling endosomes into recycling pathways, resulting in the concomitant cessation of cAMP production and the establishment of the retromer complex as a molecular off-switch for sustained GPCR signaling^139^.

### The formation of a megacomplex: a structural basis for endosome signaling

A key structural prerequisite for sustained endosomal signaling is the megacomplex (or super-complex), a ternary assembly of a GPCR, G protein, and β-arrestin. Conceptually, megacomplexes resolve the long-standing paradox of how G protein signaling can persist despite β-arrestin engagement. While the tail-engaged conformation, which leaves the TMD accessible to the G protein, is now recognized as a permissive architectural feature for megacomplex formation, the precise geometry and stability of GPCR–β-arrestin–G protein assemblies were initially unclear^29,130^.

Direct evidence for megacomplex formation was first provided using a β2V2R chimera (β_2_AR fused to the V2R C-terminal tail)^42,64^. Thomsen et al. biochemically demonstrated that a single receptor species can form a ternary assembly with both heterotrimeric G protein and β-arrestin and mediate sustained G protein signaling in cells; notably, they presented representative negative-stain EM images of the formation of megacomplexes^64^. Subsequently, Nguyen et al. resolved the structure of the β2V2R‒G_s_‒βarr1 megacomplex, revealing that the high-affinity phosphotail of V2R locks β-arrestin into a specific tail-engaged conformation while the intracellular core remains fully accessible to the G_s_ heterotrimer^42^ (Fig. 6b–d). This engagement is driven by a cluster of six specific phosphorylated residues on the V2R tail (pS357, pT359, pT360, pS362, pS363, and pS364), which form extensive electrostatic networks with conserved lysine/arginine clusters on the βarr1 N-domain^42^ (Fig. 6d). Crucially, molecular dynamics simulations further revealed that this “hanging” geometry is stabilized by the C-terminal loops of βarr1 (Fig. 6b), which make transient membrane contacts that orient βarr1 upright and prevent core engagement, thereby leaving the intracellular receptor cavity accessible for G_s_ coupling (Fig. 6c).

Although early structures relied on chimeric phosphotails, the recent cryo-EM structure of the β_2_AR_3muts (K^6.35^R, G^6.38^S, and I^6.39^V)‒G_s_‒βarr1 complex has redefined this paradigm by demonstrating that class A GPCRs can also form megacomplexes under appropriate allosteric conditions^68^ (Fig. 6e–g). Unlike tail-engaged models dominated by N-lobe engagement, this assembly adopts a C-lobe-dominant binding mode that is independent of the phosphorylated receptor C-tail. Here, βarr1 associates with the receptor primarily through C-lobe membrane anchoring, which is stabilized by the allosteric modulator atazanavir bridging the TM6/7 region of the receptor and the βarr1 197-loop^68^ (Fig. 6g). Structurally, βarr1 adopts a vertical “pendulum” conformation that occupies the interfacial groove between the Gα and Gβγ subunits, allowing it to align compatibly with the heterotrimer across multiple nucleotide-binding states (Fig. 6e). Conceptually, this structure identifies atazanavir as a molecular glue that converts β-arrestin from a competitive inhibitor of G protein coupling into a cooperative scaffold, highlighting a therapeutic strategy in which allosteric ligands stabilize noncanonical β-arrestin states to sustain signaling.

## Physiological imperatives and therapeutic frontiers

The elucidation of the GPCR–arrestin structural multiverse has reframed arrestins as central organizers of physiological signaling rather than passive terminators of G protein activity. Across tissues, arrestins function as tunable, spatiotemporal integrators that coordinate the signal strength, duration, and localization^107,141^. Consequently, physiological homeostasis depends not only on receptor activation but also on the precise engagement, conformation, and lifetime of GPCR–arrestin complexes. The disruption of this balance through altered phosphorylation patterns, impaired arrestin recruitment, or mislocalized signaling emerges as a unifying mechanism underlying diverse human diseases^142–144^.

### Clinical manifestations of an arrestin–G protein imbalance

Many clinical pathologies arise from distorted coupling between G proteins and arrestins rather than simply from receptor hyperactivity. In cardiovascular disease, chronic catecholamine exposure drives excessive beta-1 adrenoceptor (β_1_AR) phosphorylation, leading to receptor downregulation and maladaptive cardiac remodeling^145^. The clinical efficacy of carvedilol exemplifies how the selective engagement of β-arrestin microstates can restore balance: by suppressing toxic G_s_/cAMP signaling while preserving a cardioprotective β-arrestin-EGFR-ERK axis, carvedilol improves outcomes despite attenuated canonical signaling^146–148^.

In the nervous system, the MOR highlights a different dimension of imbalance. Here, G protein signaling mediates analgesia, whereas βarr2 recruitment has been implicated in the adverse effects of opioids, including respiratory depression, constipation, and tolerance^149,150^. Importantly, accumulating evidence suggests that these outcomes do not arise simply from β-arrestin recruitment per se but rather from the stabilization of specific β-arrestin conformations and signaling complexes downstream of MOR^151–153^. Therapeutic strategies that indiscriminately suppress arrestin engagement may therefore be suboptimal, highlighting the need for ligands that selectively modulate arrestin signaling states rather than globally eliminating arrestin coupling.

Endocrine disorders further illustrate how the loss of arrestin regulation disrupts physiological timing. Skeletal homeostasis depends on tightly controlled PTH1R signaling. Constitutively active receptor mutations (Jansen’s metaphyseal chondrodysplasia, H223R^154^) drive persistent G_s_ activation, whereas C-terminal truncations (Eiken syndrome, R485X^155,156^) abolish the phosphorylation barcode required for β-arrestin recruitment. In both cases, a failure to engage β-arrestin-mediated desensitization traps PTH1R at the PM, resulting in unchecked surface cAMP signaling. Together, these examples illustrate a shared principle: disease emerges from the inappropriate persistence, localization, or geometry of GPCR–arrestin signaling complexes.

### Refining therapeutic inputs: from ligand bias to structural control

Early efforts to exploit biased signaling focused on orthosteric ligands designed to favor either G protein or arrestin pathways. While conceptually transformative, this strategy has proven difficult to translate to the clinic. The G protein-biased MOR agonist oliceridine (TRV130) was developed to retain analgesic effects while reducing β-arrestin-associated side effects^157^. Although oliceridine successfully minimized β-arrestin recruitment, its clinical benefit was more modest than anticipated, suggesting that biased signaling outcomes may be context dependent and likely rely on the precise modulation of specific β-arrestin microstates rather than global pathway suppression^158–160^. Similarly, the β-arrestin-biased angiotensin II type 1 receptor (AT1R) ligand TRV027 showed promise in preclinical models but failed to outperform the placebo in phase II trials of patients with heart failure, underscoring the challenge of extrapolating system-level bias across species and disease contexts^161–163^. These outcomes highlight a key limitation of first-generation biased ligands: they modulate signaling indirectly from the extracellular orthosteric site, lacking precise control over the intracellular transducer interface. However, the clinical efficacy of carvedilol in heart failure patients demonstrates that therapeutic success extends beyond simple binary pathway selection. By simultaneously antagonizing G_s_/cAMP signaling and stimulating cardioprotective β-arrestin signaling^148^, carvedilol exemplifies the need for nuanced signal integration. Consequently, rational drug design should evolve from empirical pathway suppression toward precise structural control, directly targeting the intracellular interface to stabilize specific, therapeutically beneficial receptor–transducer microstates.

A decisive conceptual advance has emerged with the development of IAMs, facilitated by the growing number of resolved structures depicting GPCR–transducer interaction interfaces. Unlike orthosteric ligands, IAMs act directly on the intracellular pocket to enforce defined coupling geometries. The small molecule PCO371 binds within the intracellular cavity of PTH1R, stabilizing a Gα_s_-compatible conformation while sterically hindering β-arrestin engagement, thereby sustaining signaling and disfavoring internalization^122,123^. Conversely, SBI-553 occupies the Gα_q_ binding pocket of NTSR1, simultaneously blocking G protein coupling and inducing a noncanonical β-arrestin loop-engaged conformation^24,33,91^. While conventional allosteric modulators can drive signaling bias by inducing receptor-wide conformational changes similar to those induced by orthosteric agonists, these distinct IAMs show that signaling bias can also be dictated directly at the transducer interface.

In addition to modulating the receptor conformation, recent discoveries reveal that β-arrestin itself can be directly reprogrammed. The atazanavir-stabilized GPCR–G protein–βarr1 megacomplex exemplifies a new therapeutic paradigm in which small molecules function as molecular glues^68^. By bridging the βarr1 C-lobe to the receptor, atazanavir converts βarr1 from a competitive antagonist of G protein coupling into a cooperative signaling scaffold. Unlike traditional biased agonists, which struggle to completely abolish β-arrestin recruitment, this noncanonical “pendulum” conformation allows G protein and β-arrestin to coexist on the receptor^68^. By circumventing the steric competition that typically drives receptor desensitization, this molecular glue strategy sustains therapeutic G protein signaling even in the presence of β-arrestin, offering a direct mechanistic solution to drug tolerance.

Together, intracellular modulators and molecular glues extend the structural multiverse framework from descriptive biology to actionable pharmacology. Rather than targeting receptors alone, next-generation therapeutics can selectively sculpt arrestin microstates, manipulate the signaling geometry, and rewire the spatiotemporal signaling logic. This shift from ligand bias to structural control of transducer assemblies positions arrestin-centered pharmacology as a powerful frontier for achieving durable efficacy with reduced side effects.

## Concluding remarks and future directions

Advances in cryo-EM and complementary structural approaches have fundamentally reshaped our understanding of GPCR–arrestin coupling. What was once viewed as a simple binary interaction — receptor on, arrestin off — has now emerged as a continuous, context-dependent spectrum of conformational states. High-resolution structures captured in native-like membrane environments reveal that arrestin can engage GPCRs through deep-core insertion, shallow tail tethering, and a range of intermediate loop-, side-engaged, and membrane-anchored conformations. Collectively, these architectures establish that arrestin recruitment is not exclusively synonymous with signal termination but instead encodes graded functional outputs through discrete conformational microstates.

A central conclusion of this review is that arrestin functions as a programmable signal integrator rather than a passive adapter. Arrestin isoforms decode identical phosphorylation inputs differently through variations in the C-edge architecture, membrane anchoring, lipid sensitivity, and conformational plasticity, yielding isoform-selective scaffolding outcomes. The GPCR class further constrains this decoding logic: class A receptors favor dynamic 1:1 assemblies that toggle between desensitizing and scaffolding states; class B1 receptors stabilize tail-engaged conformations compatible with megacomplex formation and sustained endosomal signaling; and obligate dimeric class C receptors enforce shallow, peripheral arrestin interfaces that bias toward scaffolding rather than steric G protein exclusion. Thus, arrestin signaling emerges from the intersection of receptor topology, isoform mechanics, and cellular context.

The phosphorylation barcode provides a dominant, but not exclusive, layer of control over arrestin engagement. The motif composition and spatial arrangement — rather than merely the absolute number of phosphates — govern arrestin activation by selectively disrupting autoinhibitory restraints and promoting interdomain rearrangements. These barcodes are actively written by GRKs, whose isoform-specific localization and kinetics determine whether arrestin engagement is transient or long-lived. Superimposed on this code is a lipid-dependent regulatory layer in which membrane phosphoinositides and soluble inositol phosphates act as allosteric cofactors that prime, stabilize, and spatially restrict arrestin activation. Together, phosphorylation and lipid codes define a multi-dimensional regulatory logic that governs arrestin microstate selection.

These structural principles provide a mechanistic foundation for biased signaling that extends beyond ligand efficacy alone. Biased agonism arises from selective stabilization of receptor–transducer geometries, often through subtle rearrangements outside the canonical transducer-binding pocket. IAMs and molecular glue exemplify this concept by directly reshaping the transducer interface and converting β-arrestin from a competitive terminator into a cooperative signaling scaffold. These mechanisms underscore that bias is a geometrically encoded property of the receptor–transducer complex, not merely a pharmacological abstraction.

Spatial regulation represents a further critical dimension of arrestin biology. The discovery of GPCR–G protein–β-arrestin megacomplexes and sustained endosomal signaling challenges the long-standing view that arrestin solely terminates G protein signaling at the PM. Instead, β-arrestin can stabilize G protein signaling-competent complexes within endosomes, encoding temporal and spatial information essential for transcriptional and physiological responses. How distinct β-arrestin microstates are selectively generated, maintained, and terminated across cellular compartments remains a central unanswered question.

Looking forward, several priorities emerge for the field. First, capturing arrestin dynamics requires integrating time-resolved cryo-EM to capture millisecond intermediates with single-molecule FRET and molecular dynamics simulations spanning microsecond-to-second timescales. Second, expanding structural coverage to underexplored GPCR classes, orphan receptors, and βarr2-specific complexes will test the generality of current models. Third, linking defined structural microstates to in vivo physiology remains a major challenge; a combination of structure-guided perturbations with genetically encoded biosensors and spatially resolved signaling assays is needed to bridge this gap. Finally, the translation of the structural logic of arrestins into therapeutics will require precision strategies that target not only receptors but also GRKs, lipids, and arrestin interfaces themselves.

In summary, arrestins should now be viewed as programmable, spatiotemporal integrators of GPCR signaling that operate across conformational, biochemical, and spatial dimensions. Decoding how regulatory inputs sculpt arrestin microstates provides a unifying framework for interpreting GPCR physiology and a roadmap for designing next-generation therapeutics that exploit signaling bias, endosomal signaling, and arrestin-directed modulation with unprecedented precision.
